# *Agrobacterium tumefaciens* responses to plant-derived signaling molecules

**DOI:** 10.3389/fpls.2014.00322

**Published:** 2014-07-08

**Authors:** Sujatha Subramoni, Naeem Nathoo, Eugene Klimov, Ze-Chun Yuan

**Affiliations:** ^1^Southern Crop Protection and Food Research Centre, Agriculture & Agri-Food CanadaLondon, ON, Canada; ^2^Department of Biology, University of Western OntarioLondon, ON, Canada; ^3^Department of Microbiology and Immunology, University of Western OntarioLondon, ON, Canada

**Keywords:** *Agrobacterium tumefaciens*, virulence, signaling mechanism, gene regulation, quorum sensing

## Abstract

As a special phytopathogen, *Agrobacterium tumefaciens* infects a wide range of plant hosts and causes plant tumors also known as crown galls. The complexity of *Agrobacterium*–plant interaction has been studied for several decades. *Agrobacterium* pathogenicity is largely attributed to its evolved capabilities of precise recognition and response to plant-derived chemical signals. *Agrobacterium* perceives plant-derived signals to activate its virulence genes, which are responsible for transferring and integrating its Transferred DNA (T-DNA) from its Tumor-inducing (Ti) plasmid into the plant nucleus. The expression of T-DNA in plant hosts leads to the production of a large amount of indole-3-acetic acid (IAA), cytokinin (CK), and opines. IAA and CK stimulate plant growth, resulting in tumor formation.* Agrobacterium* utilizes opines as nutrient sources as well as signals in order to activate its quorum sensing (QS) to further promote virulence and opine metabolism. Intriguingly, *Agrobacterium* also recognizes plant-derived signals including γ-amino butyric acid and salicylic acid (SA) to activate quorum quenching that reduces the level of QS signals, thereby avoiding the elicitation of plant defense and preserving energy. In addition, *Agrobacterium* hijacks plant-derived signals including SA, IAA, and ethylene to down-regulate its virulence genes located on the Ti plasmid. Moreover, certain metabolites from corn (*Zea mays*) also inhibit the expression of *Agrobacterium* virulence genes. Here we outline the responses of *Agrobacterium* to major plant-derived signals that impact *Agrobacterium*–plant interactions.

## INTRODUCTION

*Agrobacterium* is a genus of Gram-negative bacteria that uses horizontal gene transfer to cause tumors in many plant species with agricultural and economic importance including woody ornamental shrubs (rose), vines (grape), shade trees, fruit trees (cherry, berry, walnut), and herbaceous perennials. *Agrobacterium tumefaciens* is the most commonly studied species in this genus. *A. tumefaciens* causes typical crown-gall diseases. The disease manifests as a tumor-like growth or gall usually at the junction of the root and shoot. Infection by the species *Agrobacterium vitis* results in cane gall on grapevines while *A. rhizogenes* causes excessive formation of hairy roots or root tumors. *Agrobacterium*–plant interaction is an excellent paradigm for studying both plant and bacterial responses, as well as the role of chemical signaling in these processes. *A. tumefaciens*–plant interaction is now relatively well-understood as a result of significant findings made over the past four decades (for reviews refer to Gelvin, 2003; Brencic and Winans, 2005; McCullen and Binns, 2006; Yuan and Williams, 2012; Pitzschke, 2013). The virulence proficiency of *A. tumefaciens* is dependent on the presence of the Tumor-inducing (Ti) plasmid, which harbors a Transferred DNA (T-DNA) defined by two direct repeat sequences of approximately 25 base pairs, termed the left and right borders. Most studies have made use of nopaline metabolizing strains C58 and T37 (carrying plasmids pTiC58 and pTiT37, respectively) or the octopine utilizing strain A6 (carrying pTiA6). As a ubiquitous soil bacterium, *Agrobacterium* is capable of two lifestyles: independent free-living or acting as a pathogen in association with a plant host. When living independently, *Agrobacterium* virulence is essentially silent. Upon detection of plant-derived signals in the rhizosphere, *Agrobacterium* activates its chromosomal virulence genes (*chv* genes) and Ti plasmid encoded virulence genes (*vir* genes). *Vir* genes are directly involved in T-DNA cleavage from the Ti plasmid, T-DNA processing, transferring and integration into plant nuclei, conversely, *Chv* genes are not directly involved in the T-DNA transfer process. Instead, *chv* genes play important roles in signal transduction necessary for *Agrobacterium* pathogenicity. Since T-DNA carries genes for the synthesis of indole-3-acetic acid (IAA) and cytokinin (CK; also called oncogenic genes), their expression in plants leads to the production of a large amount of plant hormones that promote uncontrolled cell division and undifferentiated growth of plant tissues, resulting in the formation of a plant tumor and permanent plant genetic transformation.

In addition to genes responsible for IAA and CK production, T-DNA also contains genes for the synthesis of opines (unusual amino acid and sugar condensates). Opines produced by transformed plant cells can be metabolized by *Agrobacterium* as a source of nutrients. In addition, opines act as signals that activate *Agrobacterium* quorum sensing (QS). QS is a special form of cell-to-cell communication by which microorganisms synthesize, release, and perceive diffusible signals such as *N*-(3-oxooctanoyl)-DL-homoserine lactone (3OC8-HSL). QS enables a single cell to sense the number of surrounding cells (cell density) and coordinates their collective behavior. In *Agrobacterium*, QS plays important roles in interaction with plant hosts, which will be discussed in later sections.

Interestingly, T-DNA encoded oncogenic genes are neither physiologically nor biologically required for the T-DNA transfer process. Therefore, T-DNA encoded genes can be deleted and replaced with genes of interest, and such genetically modified “T-DNA” can still be transferred, integrated and expressed in the plant cell. This unique ability of inter-kingdom DNA transfer makes *Agrobacterium* an important tool for genetically modifying plants, allowing for incorporation of useful traits like resistance to insects and herbicides, production of recombinant vaccines, proteins, etc. In addition, T-DNA is distally located from *vir* genes required for T-DNA transfer process. Thus, T-DNA and *vir* genes can be separated onto two plasmids without affecting T-DNA transfer into plant hosts. This feature prompted the design and construction of binary vectors that greatly facilitate DNA manipulation and plant transformation, especially considering the large size of the Ti plasmid (over 200 kb).

*Agrobacterium* is capable of infecting/transforming a wide variety of plant species including long-lived woody plants and cultivated plants. However, plants vary greatly in their ability to be infected/transformed by *Agrobacterium*, even among ecotypes within species, and the underlying molecular mechanisms are poorly understood (Nam et al., 1997). To mount a successful infection in nature, it is important for *Agrobacterium* to precisely and specifically recognize and respond to a combination of plant-derived signals in the rhizosphere including acidity, plant released sugars and plant-derived phenolic compounds (Stachel et al., 1985; Brencic and Winans, 2005; McCullen and Binns, 2006; Gelvin, 2012; Yuan and Williams, 2012; Pitzschke, 2013). *Agrobacterium* virulence programing and associations with plant hosts are stringently and synergistically regulated by a combination of plant-derived chemicals.

## *Agrobacterium* RESPONSES TO ACIDIC SIGNALS CAUSED BY PLANT-DERIVED CHEMICALS IN THE RHIZOSPHERE

The rhizosphere is the narrow region (within millimeter range of roots) of soil that is directly influenced by root exudates and is densely populated by soil microorganisms. Rhizosphere is rich in not only plant-derived but microbe-derived signals as well (Winans, 1992; Phillips et al., 2004; Bais et al., 2005). Plants routinely secrete organic acids such as lactic, citric, oxalic, and malic acids as well as other secondary metabolites, resulting in acidic rhizosphere conditions (Rivoal and Hanson, 1994; Xia and Roberts, 1994; Walker et al., 2003; Phillips et al., 2004; Bais et al., 2005; Wang et al., 2006; Huckelhoven, 2007; Badri and Vivanco, 2009). Upon wounding, plants release phenolic compounds as well as neutral and acidic sugars necessary to repair damaged tissue acidifying the rhizosphere (Winans, 1992). Therefore the rhizosphere, where *Agrobacterium* primarily infects plant hosts, is typically an acidic niche driven by various plant-released chemicals.

Upon close proximity to a suitable plant host in the rhizosphere, acidic conditions and plant-derived chemicals play important roles in initiating the *Agrobacterium* virulence program, which involves various *Agrobacterium* regulatory factors and signaling pathways (Winans, 1992). A chromosomally encoded *che* cluster (chemotaxis) allows *A. tumefaciens* to be attracted to plant-derived chemicals in the rhizosphere (Wright et al., 1998). In addition, three *Agrobacterium* chromosomally encoded genes *chvA*, *chvB*, and *exoC* are involved in synthesis of extracellular oligosaccharides, such as cyclic 1,2-b-D-glucan, that allows* Agrobacterium* to attach to plant hosts (Cangelosi et al., 1989). Upon perception of acidity characteristic of the rhizosphere, *Agrobacterium* mounts both a conserved response as well as a signaling specific response to infect plant hosts. This conserved response allows *Agrobacterium* to adapt to the rhizosphere niche by modulating metabolism and cellular adaptation, such as the induction of genes coding for cell envelope synthesis, stress response, transporters of sugars and peptides (Yuan et al., 2008b).

The signaling specific response to acidity is mediated by the chromosomally encoded ChvG/ChvI two-component system, as well as other genes that allow *Agrobacterium* to initiate its early virulence program (Yuan et al., 2008a). ChvG acts as the sensor kinase while ChvI functions as the response regulator (Winans, 1990, 1992; Chen and Winans, 1991; Charles and Nester, 1993; Mantis and Winans, 1993; Li et al., 2002). The ChvG/I system is believed to recognize acidity in the rhizosphere and activates the expression of several virulence factors including *chvI*, *aopB* encoding an outer membrane protein, *katA* encoding a catalase, *pckA* encoding phosphoenol carboxykinase, and the *imp* gene cluster encoding a type VI secretion system (T6SS; Yuan et al., 2008a). A more recent study confirmed that *Agrobacterium* T6SS is indeed induced by acidity in a ChvG/ChvI dependent manner (Wu et al., 2012). Perhaps most interestingly, it was found that upon perception of acidic signals, several *vir* genes were also induced including *virG*, *virE0*, and *virH* (Yuan et al., 2008a), consistent with the observation that the ChvG/ChvI system activates the proximal promoter (P2) of *virG* (Li et al., 2002). However, to be functional, VirG requires phosphorylation signaling from another plant-derived signal, e.g., plant-derived phenolic compounds, which will be discussed in the following section. It is noteworthy that in addition to ChvG/I, another chromosomally encoded virulence gene, *chvE*, is also involved in *Agrobacterium* response to acidity and plant-derived sugars in the rhizosphere, which will also be discussed later.

## *Agrobacterium* RESPONSES TO PLANT-DERIVED PHENOLIC COMPOUNDS

Originally it was believed that plant wounding was necessary for *Agrobacterium* infection and pathogenicity. However, recent advances have found that plant wounding is in fact not essential for *Agrobacterium* pathogenicity since unwounded plants can also infected by *Agrobacterium* pathogens (Brencic and Winans, 2005). Besides acidic signals, plant-derived phenolic compounds are essential for the induction of *Agrobacterium* virulence (Stachel et al., 1986). Moreover, phenolics serve as chemoattractants for *Agrobacterium* (Parke et al., 1987; Melchers et al., 1989). Structural specificities of virulence inducing phenolics include the presence of a benzene ring with a hydroxyl group at position 4 and a methoxy group at position 3 (Dixon and Paiva, 1995). 3,5-dimethoxyacetophenone (acetosyringone) and hydroxyacetosyringone were the first identified inducers of *Agrobacterium* virulence (Stachel et al., 1985; Hess et al., 1991). The *Agrobacterium* VirA/VirG two-component system located on the Ti plasmid has been suggested to recognize acetosyringone as a host specific signal and activate *vir* gene expression (Winans et al., 1986; Leroux et al., 1987; Shaw et al., 1988; Winans, 1990). The membrane receptor VirA functions as a dimer with four domains; the periplasmic, cytoplasmic linker, kinase, and receiver domains. Upon phenolic signal perception, the linker domain of one VirA subunit activates the kinase domain of the opposite dimerized subunit by intermolecular phosphorylation (Chang and Winans, 1992; Turk et al., 1994; Toyoda-Yamamoto et al., 2000). However, a previous study has shown the binding of radiolabelled phenolic compounds to two small proteins other than VirA and controversy remains regarding the exact mechanism involved in phenolic detection by VirA (Lee et al., 1992). Nevertheless, the auto-phosphorylated sensor kinase VirA phosphorylates the cytosolic response regulator VirG at the conserved Asp52 (Morel et al., 1990; Lee et al., 1995). Phosphorylated VirG binds to a 12-bp *vir* box located upstream of transcription start sites of *vir* genes, thereby activating their transcription (Stachel et al., 1985; Stachel and Nester, 1986; Jin et al., 1990a,b,c; Pazour and Das, 1990; Roitsch et al., 1990). In fact, phosphorylated VirG also activates its own expression by activating *virG* transcription at the distal promoter (p1; Chang and Winans, 1992; Liu et al., 1992, 2005; Jia et al., 2002; Li et al., 2002; Yuan et al., 2008a; Wise et al., 2010).

*Vir* genes of *Agrobacterium* are organized in several *vir* operons. There are eight *vir* operons on the octopine-type Ti plasmid and relatively fewer *vir* genes on the nopaline-type Ti plasmid (Stachel and Nester, 1986; Rogowsky et al., 1987; Kalogeraki and Winans, 1998; Kalogeraki et al., 2000; Brencic and Winans, 2005). The *vir* operons are typically organized as *virH*,* virA*,* virB*,* virG*,* virC*,* virD*,* virE*, and *virF* transcriptional units (Brencic and Winans, 2005). *Vir* genes code for a set of proteins with different functions such as T-DNA excision and processing (*virC* and *virD*), coating and protecting T-DNA during transfer (*virE*), formation of the type IV secretion system (T4SS) responsible for the delivery of T-DNA to plant cells (*virB operon*), and T-DNA integration into plant nucleus (*virE2* and *virD4*). A study by Cho and Winans (2005) revealed that each gene on the Ti plasmid was modestly induced by plant-derived phenolic signals, while the *repABC* operon, responsible for Ti plasmid replication/partitioning, was significantly induced by phenolic signals. This suggests that the copy number of the Ti plasmid is induced by plant-derived phenolics, which is confirmed by direct binding of phosphorylated VirG to a 12-bp *vir* box upstream of the *repABC* operon (Zhu et al., 2000; Pappas and Winans, 2003; Cho and Winans, 2005). Apparently an increase in Ti plasmid copy number enhances the dosage of* vir* genes responsible for T-DNA transfer. A proteomic study corroborated VirA/VirG dependent induction of *vir* genes by identifying 11 proteins that were significantly induced in response to acetosyringone, including proteins constituting the T4SS, the single strand binding protein VirE2 that is exported to the plant nucleus, and the trans-zeatin synthesizing protein Tzs (Lai et al., 2006). Moreover, responses to phenolic inducers may be modulated by detoxification of these compounds by VirH2. VirH2 was shown to play a role in the metabolism of several phenolic compounds including ferulic acid, another inducer of *vir* genes (Brencic et al., 2004).

## *Agrobacterium* RESPONSES TO PLANT RELEASED SUGARS IN THE RHIZOSPHERE

*Agrobacterium* detects and responds to plant-derived sugars through a distinct signaling pathway involving VirA and a chromosomally encoded periplasmic protein, ChvE (Cangelosi et al., 1990). Expression of c*hvE* is regulated by the LysR transcriptional regulator (TraR) galactose-binding protein regulator (GbpR) in the presence of sugars (Doty et al., 1993; Peng et al., 1998). ChvE mediates *Agrobacterium* chemotaxis in response to aldose monosaccharides such as galactose, glucose, arabinose, fucose, xylose, and sugar acids. Importantly, ChvE binds plant-derived sugars and subsequently interacts with the periplasmic domain of VirA to stimulate *vir* gene expression (Cangelosi et al., 1990; He et al., 2009; Hu et al., 2013). Mutations in the periplasmic domain of VirA present the same phenotype as a ChvE mutant with both mutants unable to infect specific plant hosts (Cangelosi et al., 1990; Chang and Winans, 1992; Banta et al., 1994; Peng et al., 1998; Gao and Lynn, 2005). Recent studies also suggest that the ability of ChvE to recognize and bind different plant-derived sugars is vital in determining the host range of *Agrobacterium* (Hu et al., 2013). Interestingly, the sugar response in *Agrobacterium* has been found to be linked with the acidity responses since the absence of sugars or mutations in *chvE* disrupted acidic signaling. In addition, the affinity of ChvE for sugar acids increases with a decrease in pH (Hu et al., 2013), which reinforces an important role for acidity in modulating *Agrobacterium* virulence. It has been proposed that acidic conditions, together with the presence of sugars and a functional ChvE, promotes VirA–ChvE interactions required for efficient *vir* gene induction (Shimoda et al., 1993; Toyoda-Yamamoto et al., 2000; Gao and Lynn, 2005; Nair et al., 2011). However, mutations in *chvE* that abolish sugar sensing do not abolish *vir* gene induction by acetosyringone, although ChvE is known to interact with the periplasmic domain of VirA. This suggests ChvE and associated sugar perception play additive roles that further promote *vir* gene expression in response to sugars and phenolic compounds, while phenolics are essential *vir* gene inducing signals (Cangelosi et al., 1990; He et al., 2009; Hu et al., 2013). Apart from its signaling role, ChvE also has a role in sugar utilization as it delivers sugars to the ABC transporter MmsAB (Hu et al., 2013).

## BACTERIAL AND PLANT MOLECULES INVOLVED IN T-DNA TRANSFER AND INTEGRATION

T-DNA transfer and integration into the plant nucleus is mediated by a complex set of *Agrobacterium* and host proteins. As discussed in previous sections, *Agrobacterium* recognizes three main plant-derived signals (acidity, phenolics, and sugars) and activates *vir* genes. VirD1, a helicase, and VirD2, a site specific endonuclease, are essential for nicking the Ti plasmid and the release of T-DNA as a single stranded DNA (referred to as T-strand; Yanofsky et al., 1986; Wang et al., 1990). VirD4, the coupling protein, and VirB1–VirB11, the mating-pore-formation components, together constitute the trans-envelope channel and pilus of *Agrobacterium* T4SS apparatus. In depth studies have assigned functional roles to each protein of the T4SS complex; VirD4, VirB3, VirB4, and VirB11 constitute the ATP-dependent translocation machinery, VirB6–VirB10 form the channel and VirB1, VirB2, VirB5, and VirB7 form the pilus (Cascales and Christie, 2003). The T-strand is covalently attached by VirD2, which is subsequently bound by VirD4 and VirB11 forming the T-complex. Current findings suggest that such DNA binding by associated virulence proteins (VirD4 and VirB11) stimulates ATP hydrolysis to produce a structural transition in the membrane channel protein VirB10. This allows passage of the T-complex to the cell surface where it can be directed to the T4SS pili, followed by delivery into the plant cell by the T4SS apparatus (Cascales and Christie, 2003; Cascales et al., 2013). Notably, many Gram-negative plant and animal pathogenic bacteria employ a type III secretion system (T3SS) to inject effector proteins directly into the cytosol of eukaryotic cells and thus allow the manipulation of host cellular activities to the benefit of the pathogen (Buttner and He, 2009). However, no T4SS organelles in *Agrobacterium* reminiscent of the basal body of flagella or needle complexes of the T3SS were evident (Christie, 2004). The exact process is still unknown regarding how the T-DNA complex is delivered by T4SS into plant cells, especially how the T-DNA complex passes through cell wall and plasma membrane, subsequently moving through the cytoplasm to the plant cell nucleus (Cascales and Christie, 2003; Gelvin, 2003).

T-DNA integration into plant nuclei is thought to occur by hijacking various host systems including defense signaling, cytoskeletal networking, molecular motors, nuclear import, proteolytic degradation, chromatin targeting, and DNA repair to ensure successful plant transformation (for review see Citovsky et al., 2007). In the plant cytoplasm, the T-strand/VirD2 complex is coated along its entire length by the VirE2 ssDNA-binding protein that is transported into the plant cell independently of the T-strand complex (Vergunst et al., 2000; Citovsky et al., 2007). Both VirD2 and VirE2 carry plant nuclear localization signals and together with host protein VIP1 (VirE2 interacting protein 1), facilitate T-complex import into the plant cell for host chromatin targeting (Citovsky et al., 1992; Tzfira et al., 2001; Li et al., 2005; Djamei et al., 2007; Lacroix et al., 2008). T-DNA is thought to attach to chromatin by interacting with nucleosomal proteins and is released from the T-complex by proteolytic removal of associated proteins (Magori and Citovsky, 2011). VirF, a bacterial F-box protein, also targets both VIP1 and VirE2 for proteasome dependent degradation. The mechanism of T-DNA integration into the plant genome is thought to occur by illegitimate recombination; however, the details of many of the molecular events within the plant cell and nucleus are still unclear.

## *Agrobacterium* METABOLIZES PLANT-DERIVED OPINES AS A SOURCE OF NUTRIENTS

Besides IAA and CK, infected plant cells produce over 20 different kinds of opines that can be classified into four families: octopine, nopaline, mannopine, and agrocinopine families (Beck von Bodman et al., 1992; Fuqua and Winans, 1996b; Piper et al., 1999). In fact, the most intensively studied Ti plasmids are the octopine and nopaline types, named after the predominant opines synthesized by transformed plant cells. Octopine is synthesized by the T-DNA-encoded enzyme octopine synthase (Ocs), which condenses pyruvate with different amino acids to produce octopine, lysopine, histopine, or octopinic acid (Dessaux et al., 1998). Nopaline is generated by nopaline synthase (Nos) in a similar condensation reaction involving αα-ketoglutaric acid and either arginine or ornithine. Opines of the mannopine and agrocinopine families are structurally more heterogeneous, which contain sugar and phosphate groups in the case of agrocinopine. Since plants cannot metabolize opines, transformed plant cells accumulate and release opines into the rhizosphere. The precise mechanism by which opines are exuded from plant cells is unknown, although the exudation of octopine and nopaline appears to depend upon the product of T-DNA gene 6a. Nevertheless, opines are present on the plant (or tumor) surface and are part of the soluble plant exudates released into the phylloplane and rhizoplane (Savka and Farrand, 1992).

*Agrobacterium* Ti plasmids also contain genes for opine uptake and catabolism that are located in the non-transferrable region, e.g., occ and noc regions for octopine- and nopaline-type Ti plasmids. In addition, Ti plasmids contain chemotaxic genes for their corresponding opines (Beck von Bodman et al., 1992; Kim and Farrand, 1997). *Agrobacterium* LysR-type transcriptional activator OccR (octopine catabolic regulator) and NocR (nopaline catabolic regulator) recognize and bind to opines, subsequently activating the expression of opine catabolic genes (Beck von Bodman et al., 1992; Wang et al., 1992). *Agrobacterium* metabolism of agrocinopine is much more complicated. When agrocinopines are present, the repressor agrocinopine catabolic regulator (AccR) dissociates from the promoter, allowing for expression of the *acc* operon responsible for agrocinopine metabolism. In addition, the *acc* operon is activated in response to phosphate limitation (Kim et al., 2008). Some of opine catabolic genes are also under regulation of other factors, for example the presence of certain substrates such as succinate (Hong et al., 1993). Although the rhizosphere contains species other than *Agrobacterium* that are capable of utilizing opines, they comprise of a very small minority of the bacterial population (Nautiyal and Dion, 1990). Therefore, the ability to use opines as a carbon, nitrogen, and energy source provides distinct advantages to *Agrobacterium* in the rhizosphere niche.

## *Agrobacterium* QUORUM SENSING IS ACTIVATED BY PLANT-DERIVED OPINES

In addition to serving as a nutrition source for *Agrobacterium*, opines produced by transformed plant cells also activate *Agrobacterium* QS. In fact, the original study of *Agrobacterium* QS was relevant to Ti plasmid conjugation. In soil or cultivation at temperatures greater than 30^∘^C, the Ti plasmid is rapidly lost from *Agrobacterium* populations. Once infected by *Agrobacterium*, plant cells produce opines. In addition to activating genes for opine metabolism, the NocR- or OccR-opine complex also activates a LuxR-type TraR located on the Ti plasmid (Li and Farrand, 2000). When the diffusible QS signal N-(3-oxooctanoyl)- DL-homoserine lactone (3OC8-HSL) reaches a threshold level with high population density, TraR binds to 3OC8-HSL. The TraR–3OC8-HSL complex activates *traI*, a LuxI-type 3OC8-HSL synthase, and *tra*/*trb* genes coding for a second T4SS responsible for conjugal transfer of Ti plasmids (Piper et al., 1993; Zhang et al., 1993; Fuqua and Winans, 1994; Hwang et al., 1994). Since the Ti plasmid carries genes responsible for plant infection and opine metabolism, avirulent *Agrobacterium* lacking the Ti plasmid becomes infectious and capable of opine metabolism by acquiring the Ti plasmid through conjugation. Additionally, the TraR–3OC8-HSL complex activates the *repABC* operon thereby enhancing the replication and copy number of the Ti plasmid (Fuqua and Winans, 1996a; White and Winans, 2007). TraR–3OC8-HSL complex also activates the transcription of TraM, a TraR antiactivator in both octopine- and nopaline-type strains of *Agrobacterium*. TraM further modulates QS and Ti plasmid conjugation in the rhizosphere (Hwang et al., 1999). Therefore, the initial infection and T-DNA transfer leads to the synthesis of opines in plant cells. Opines activate the *Agrobacterium* TraR/TraI QS system, which activates Ti plasmid conjugation and enhances Ti plasmid copy number (up to eightfold). This typical positive feedback regulation is advantageous for maximal infection of plant hosts and opine metabolism (Zhu and Winans, 1999, 2001; Pappas and Winans, 2003; Cho and Winans, 2005; Pinto et al., 2012).

## *Agrobacterium* QS IS FURTHER MODULATED BY OTHER PLANT-DERIVED SIGNALS

*Agrobacterium* QS is well regulated not only for QS signal production, but also for QS signal degradation, also known as quorum quenching. γ-amino butyric acid (GABA) significantly increases in wounded plant tissues and acidic conditions. GABA also accumulates in plants infected by *Agrobacterium* (Chevrot et al., 2006). In addition, proline significantly accumulates in plant tumors but neither in wounded nor healthy tissues (Deeken et al., 2006). The *Agrobacterium* proline/GABA receptor *atu2422* and ABC-transporter *braE* (*atu2427*) are required for GABA and proline uptake. GABA activates transcription of the *attKLM* operon located on the second plasmid of *Agrobacterium*. AttK is a NAD dependent dehydrogenase. AttL is a alcohol dehydrogenase and AttM, a γ-butyrolactonase, breaks down the *Agrobacterium* QS signal 3OC8-HSL. Proline is a competitive antagonist of GABA and is also taken up through the Atu2422-Bra ABC transporter system (Wachter et al., 2003; Haudecoeur et al., 2009). It was found that plants with relatively higher proline levels present bigger tumors and severe disease symptoms, whereas those with relatively high GABA attenuated *Agrobacterium* pathogenesis. This is likely a result of the pathogen’s enhanced virulence through QS that is negatively regulated by GABA (Brugiere et al., 1999). Furthermore, it was found that a short interfering RNA, AbcR1, targets the ribosome binding site of *atu2422* and negatively affects its translation (Wilms et al., 2011, 2012).

Recent studies revealed that the plant defense signal salicylic acid (SA) also activates the *attKLM* operon thereby down-regulating *Agrobacterium* QS (Yuan et al., 2007, 2008a). It was suggested that down-regulation of QS during the initial stages of infection benefits *Agrobacterium* pathogenicity, since high levels of QS signals are known to trigger a defense response in eukaryotic hosts (Ritchie et al., 2005; Wagner et al., 2007). Therefore, *Agrobacterium* QS is under tight and complex modulation by plant-derived opine, SA, and GABA to ensure optimum infection of plant hosts and to avoid the elicitation of plant defense responses by high levels of QS signals, reflecting an evolutionary advantage. In addition, quorum quenching induced by SA and GABA might function to prevent unnecessary energy expenditure after T-DNA transfer. Moreover, since the AttM lactonase has a broad substrate range, the activation of *Agrobacterium* quorum quenching by GABA and SA likely confers *Agrobacterium* a competitive advantage by degrading QS signals from unrelated competitive bacteria occupying the rhizosphere niche (Mathesius et al., 2003; Carlier et al., 2004; Chevrot et al., 2006; Yuan et al., 2007). Furthermore, induction of *attKLM* genes allows *Agrobacterium* to metabolize other plant-released compounds such as gamma-butyrolactone to produce succinic acid for the central metabolism (tricarboxylic acid cycle; Carlier et al., 2004; Chevrot et al., 2006; Chai et al., 2007).

## *Agrobacterium* VIRULENCE MODULATED BY PLANT HORMONES AND PLANT-DERIVED CHEMICALS

Plant hormones play important roles in plant defense and stress resistance. IAA and ethylene (ET) levels in plant tissues are elevated at the initial stages of infection by *Agrobacterium.* Following T-DNA integration, SA, IAA, and ET levels are elevated (Lee et al., 2009), while jasmonic acid (JA) levels were unchanged. However, in tumors, IAA and ET signaling pathways were activated, while JA and SA signaling pathways remained inactivated. Synthesis of IAA in crown gall occurs as a two-step process from tryptophan via indoleacetamide, mediated by-products of the T-DNA encoded *iaaM* and *iaaH* genes (Thomashow et al., 1986). T-DNA also carries an *ipt* gene responsible for CK synthesis. The *ipt* product condenses isopentenyl pyrophosphate and AMP to produce isopentenyl-AMP, which is later converted to CK by host enzymes. The elevated level of IAA and CK promote plant cell growth and tumor formation. It is now becoming evident that key phytohormones significantly influence *Agrobacterium* pathogenicity and tumor formation through both plant signaling pathways as well as direct modulation of bacterial processes (Veselov et al., 2003; Yuan et al., 2007; Zottini et al., 2007; Anand et al., 2008). The following sections are focussed on the effects of plant hormones on *Agrobacterium* pathogenicity, in particular, how *Agrobacterium* responds to these plant hormones.

### *Agrobacterium* RESPONSES TO INDOLE-3-ACETIC ACID (IAA)

IAA is important for plant growth and development, where its functions are mediated by the asymmetric distribution of IAA both systemically and locally (Korbei and Luschnig, 2011). IAA produced by *Agrobacterium* infected cells not only contributes to tumor growth, but also affects *Agrobacterium* pathogenicity. It was found that IAA, at 25 μM concentrations, inhibits *Agrobacterium vir* gene expression while not significantly affecting *Agrobacterium* growth. This is thought to occur by competition between IAA and phenolic inducers of *vir* genes for their interaction with the VirA/VirG two-component system (Liu and Nester, 2006). Further studies have indicated that IAA likely competes with *vir* gene inducing signals, such as acetosyringone, for association with the VirA linker domain, which is strengthened by the related molecular structures of acetosyringone and IAA. Activation of *vir* genes by acetosyringone and IAA-mediated inhibition of *vir* genes have never been genetically separated. Moreover, the inhibition of *vir* genes by IAA can be rescued by higher level of acetosyringone or incorporation of a constitutive *virA* expressing plasmid. Furthermore, IAA inhibits *Agrobacterium* growth at higher concentrations (over 50 μM) yet does not kill *Agrobacterium* (Liu and Nester, 2006). It was proposed that after successful transformation of a plant host, the synthesis of large amounts of IAA in infected plant tissues represses *vir* gene expression for energy conservation. Yet it remains unclear if the local concentration of IAA in fresh tumors reaches the inhibitory range (Liu and Nester, 2006).

### *Agrobacterium* RESPONSES TO SALICYLIC ACID (SA)

SA is a well-known phytohormone activating plant defense responses to incompatible interactions (Zottini et al., 2007). During *Agrobacterium*–plant interactions, SA produced in infected plants modulates the *Agrobacterium* virulence program by several mechanisms (Yuan et al., 2007; Lee et al., 2009). Apart from mounting plant defense responses, SA at biologically relevant concentrations (8–10 μM) limits *Agrobacterium* growth, represses *vir* gene expression, and dampens *Agrobacterium* QS as discussed in the previous section (Yuan et al., 2007, 2008b). In fact, SA inhibits all the *vir* genes including the *repABC* operon, suggesting SA likely prevents the increase of Ti plasmid copy number. This is consistent with the observation that SA-overproducing plants display recalcitrance to *Agrobacterium* infection, whereas mutant plants impaired in SA biosynthesis and accumulation are more susceptible to tumor growth (Yuan et al., 2007; Lee et al., 2009). Similar to IAA, the inhibition of *vir* gene expression by SA can be rescued by either increasing levels of acetosyringone or incorporation of a constitutive *virA* expressing plasmid. SA likely functions as an allosteric competitive inhibitor and interferes with the interaction between the kinase domain of VirA and acetosyringone since the constitutively expressed VirA activates *vir* gene expression independent of acetosyringone (Yuan et al., 2007).

### *Agrobacterium* RESPONSES TO ETHYLENE (ET)

ET, unlike other plant hormones, is a volatile hormone that affects many aspects of plant growth and development (Wang et al., 2013). ET also acts as a plant stress signal. ET signaling pathways are induced by various biotic and abiotic stresses including osmotic stress, salt stress, wounding, pathogen attack and flooding. These stress-induced ET signaling pathways have substantial roles in defense responses and disease resistance by accelerating senescence, abscission of infected organs and induction of specific defense proteins (Chang and Shockey, 1999). Plant tissues rich in ET, such as melons, are recalcitrant to *Agrobacterium* transformation, yet the cause for the transformation recalcitrance remains unclear. Thus, various strategies have been employed to reduce ET level to improve *Agrobacterium* transformation efficiency, including the application of an antisense ACC oxidase gene (pAP4), the final enzyme in the ET biosynthetic pathway. Recent studies have found that ET is another important factor modulating *Agrobacterium* virulence programing and determining crown gall morphogenesis (Nonaka et al., 2008). In particular, *Agrobacterium*-mediated genetic transformation was inhibited in ET-sensing melon but enhanced in ET-insensitive mutants. Further studies also revealed that *Agrobacterium* growth was not affected by ET, but the presence of ET at the beginning of *Agrobacterium* infection displays significant inhibitory activity on *vir* gene expression. Such inhibitory effects can be rescued by supplementation with acetosyringone, a *vir* gene inducer (Nonaka et al., 2008). Although the ET levels are up-regulated during *Agrobacterium* infection, plant genes for ET receptors and downstream signaling are not induced (Lee et al., 2009). These observations suggest that ET impacts *Agrobacterium*–plant interactions largely through its inhibitory effects on bacterial virulence programming.

### ADDITIONAL PLANT-DERIVED *vir* GENE INHIBITORS IN NATURE

In addition to the universal phytohormones SA, ET, and IAA, monocots contain special chemicals acting as natural inhibitors of *Agrobacterium* virulence. Maize, along with other monocots, are notoriously resistant to *Agrobacterium* transformation and the cause for this has been delimited to the inhibition of *Agrobacterium vir* genes (Heath et al., 1997). In particular, metabolites derived from corn seedlings (*Zea mays*) such as 2,4-dihydroxy-7-methoxy-2H-1,4-benzoxazin-3(4H)-one (DIMBOA) and 2-hydroxy-4,7-dimethoxybenzoxazin-3-one (MDIBOA) inhibit the expression of *Agrobacterium vir* genes in the presence of *vir* inducing signals (Sahi et al., 1990; Zhang et al., 2000). In addition, *Agrobacterium* mutants resistant to either DIMBOA or MDIBOA were much more effective in infecting plant hosts. Moreover, *Agrobacterium* carrying constitutively active *virA* are insensitive to MDIBOA in terms of the inhibition of *vir* genes. These observations suggest that DIMBOA and MDIBOA, similar to SA, ET, and IAA, probably affect signal perception by the VirA sensor kinase prior to the VirA/G phosphorylation signal relay events.

**FIGURE 1:**
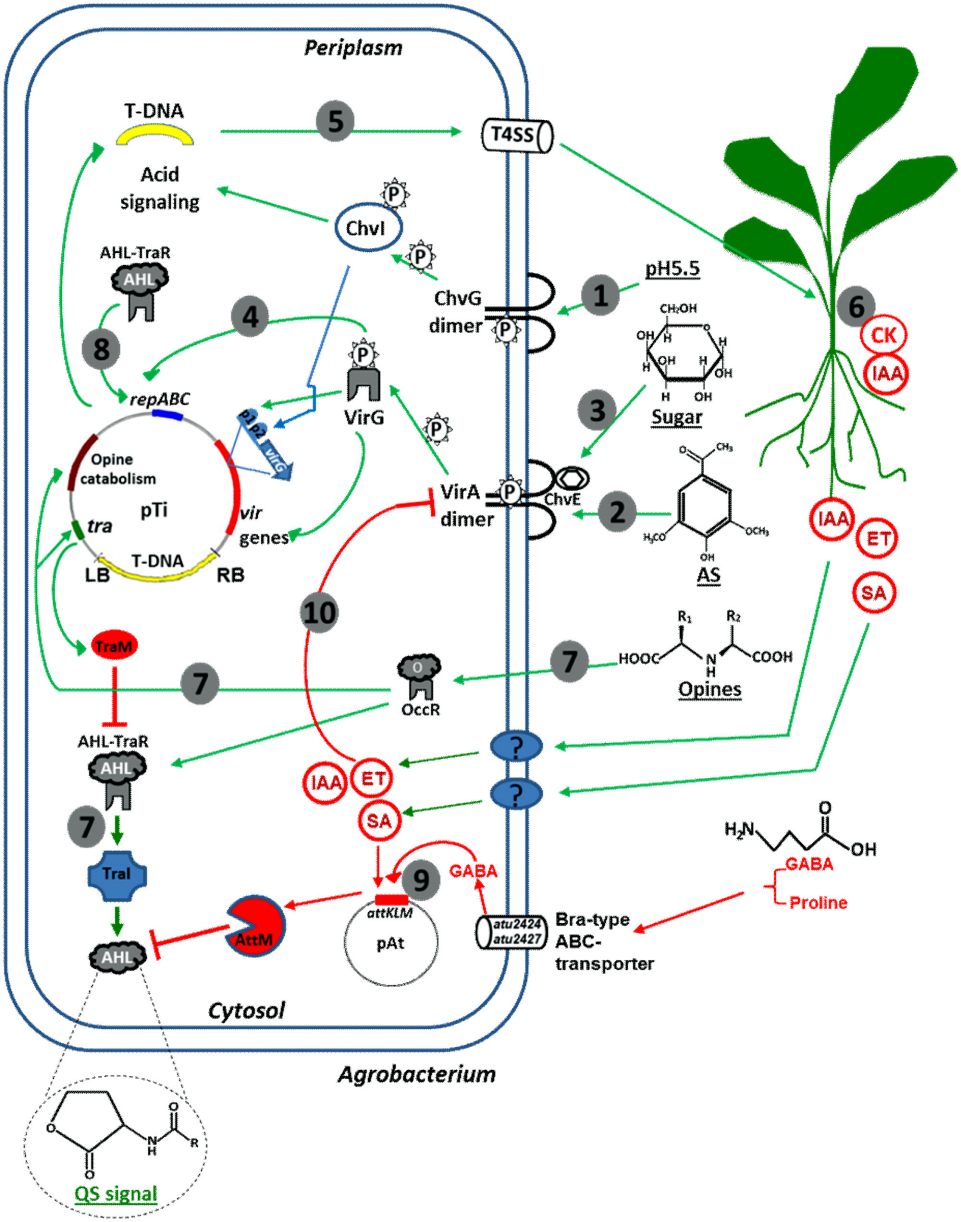
**Schematic drawing of *Agrobacterium* responses to plant-derived signals. (1)** Upon perception of acidic conditions in the rhizosphere, the ChvG/I two-component system activates the expression of several virulence genes including *chvI* and *virG*; **(2)** Upon perception of plant-derived phenolics, the VirA/G two-component system activates all *vir* genes including *virG* to further promote *vir* gene expression; **(3)** ChvE binds plant-released sugars and interacts with the VirA to allow maximal *vir* gene expression; **(4)** *Agrobacterium* Ti plasmid copy number is up-regulated in response to phenolic compounds; **(5)** *Vir* gene products process and deliver T-DNA into plant nuclei; **(6)** Expression of T-DNA encoded genes in plants leads to the production of IAA, CK, and opines; **(7)** Opine activates *Agrobacterium* genes for opine metabolism, as well as TraR/TraI QS system that subsequently induces Ti plasmid conjugation; **(8)** QS also up-regulates Ti plasmid copy number for maximal pathogenicity; **(9)** *Agrobacterium* quorum quenching (*attKLM* operon) is activated by plant-derived GABA and SA thereby down-regulates QS; **(10)** *Agrobacterium* hijacks plant-derived SA, IAA, and ET to down-regulate *vir* gene expression.

## CONCLUSION AND FUTURE PERSPECTIVES

### SIGNALING INTEGRATION AND CASCADE ACTIVATION OF *Agrobacterium* VIRULENCE BY PLANT-DERIVED SIGNALS

Several lines of evidence suggests a hierarchical activation of *Agrobacterium* virulence by a combination of plant-derived signals, as illustrated in **Figure 1**. Rhizospheric acidity activates the ChvG/I system, which subsequently activates *virG* transcription at the proximal promoter (P2) to allow basal level expression of *virG*. Therefore, the ChvG/I system functions upstream of VirA/VirG system (Li et al., 2002; Yuan et al., 2008b). Upon recognizing phenolic signals such as acetosyringone, VirA becomes auto-phosphorylated and subsequently phosphorylates VirG. Phosphorylated VirG activates the expression of *vir* genes responsible for T-DNA transfer and integration. Phosphorylated VirG also activates *virG* expression at the distal promoter (p1) to further promote virulence. ChvE binds to plant-derived sugars and subsequently interacts with the periplasmic domain of VirA to allow for the maximal expression of *vir* genes. Thus, the VirA/G system couples and integrates three rhizosphere signals: acidity, sugars (monosaccharides) and phenolic compounds. Such signaling integration and cascade activation of *Agrobacterium* virulence ensures precise perception of suitable plant hosts in the rhizosphere and maximal infection, reflecting an evolutionary advantage (Li et al., 2002; Yuan et al., 2008b). Although ChvG/I regulates VirA/G, *chvG*/*chvI* are expressed independent of the VirA/G system and plant-derived phenolic signals (Charles and Nester, 1993; Peng et al., 1998).

### PLANT-DERIVED SIGNALS FUNCTION ADDITIVELY AND PLAY REDUNDANT ROLES IN MODULATING *Agrobacterium* VIRULENCE, Ti PLASMID COPY NUMBER AND QS

Since Ti plasmid harbors *vir* genes as well as genes for opine metabolism, Ti plasmid copy number directly influences pathogenicity and the efficiency of opine metabolism. It was established that the *repABC* operon responsible for Ti plasmid replication and partition is induced by plant-derived phenolics through the VirA/G system. In addition, opine activated-QS further promotes the expression of the *repABC* operon. On the other hand, plant-derived SA and GABA activate *Agrobacterium* quorum quenching, which has negative impacts on Ti plasmid copy number. Furthermore, SA, IAA, and ET inhibit *vir* genes including the *repABC* operon, thereby preventing the increase of Ti plasmid copy number. The signaling complexity also applies to the modulation of *Agrobacterium vir* genes, which are activated by tripartite signals in the rhizosphere, acidity, phenolics, and plant-derived sugars, but down-regulated by SA, IAA, and ET and other natural *vir* gene inhibitors such as DIMBOA and MDIBOA (not shown in the **Figure 1**). In fact, the modulation of Ti plasmid copy number and conjugation also influences the overall *Agrobacterium* pathogenicity. It is noteworthy that *Agrobacterium* mounts distinct but overlapping cellular responses to SA, IAA, and GABA, despite the absence of any structural relation (Yuan et al., 2008a). Therefore, it is plausible that in nature, different plant-derived signals act in concert and function additively, playing redundant roles in tailoring *Agrobacterium* virulence, Ti plasmid copy number and QS (Yuan et al., 2008a).

### THROUGH INFECTION OF PLANTS, *Agrobacterium* CONVERTS PLANT CELL INTO A FACTORY TO SECURE NUTRIENTS AND MAINTAIN THE GENETIC INTEGRITY IN NATURE

The evolution and survival of *Agrobacterium* as a bacterial species depends on an intricate balance of two populations of cells, those which actively maintain and those which passively lose the Ti-plasmid. Both forms are necessary for the species to sustain competitive lifestyles in either the absence or presence of a plant host. In the absence of a plant host, *Agrobacterium* harboring the Ti plasmid are at a growth disadvantage to those *Agrobacterium* lacking the plasmid, which is ascribed to the metabolic burden needed to maintain such a large Ti plasmid. In the presence of a host plant and opines, the advantage is shifted to Ti plasmid-retaining *Agrobacterium* since the Ti plasmid contains genes responsible for opine uptake and metabolism. Opines not only activates genes responsible for opine metabolism, but also activate QS-dependent functions such as induction of Ti plasmid conjugation and enhancement of Ti plasmid copy number, promoting maximal infection. In fact, the increase of Ti plasmid copy number may be advantageous for Ti plasmid conjugation. Moreover, *Agrobacterium* hijacks SA and GABA signaling to activate the *AttKLM* operon which also degrades plant-derived GABA, gamma-butyrolactone, and gammahydroxy butyrate to provide even more nutrients for the tricarboxylic acid cycle. Therefore, it is reasonable to believe that through plant transformation, *Agrobacterium* converts infected plant cells into a factory to secure nutrients, in particular opines, nutrients almost exclusive for *Agrobacterium*. In addition, QS activates Ti plasmid conjugation enabling *Agrobacterium* to maintain the Ti plasmid and genetic integrity in nature.

In summary, *Agrobacterium* pathogenicity is largely attributed to its evolved capabilities of precise recognition, response to and hijacking of plant-derived chemical signals for its own benefit. The complex inter-kingdom signaling interplay and regulatory circuits highlight elegant mechanisms of *Agrobacterium*–host co-evolution. Plant roots secrete and release a wide range of chemical cues into the rhizosphere (Bais et al., 2005), admittedly, only a limited number of plant-derived chemicals have been intensively studied for their roles in *Agrobacterium*–plant interactions. For future studies, it will be worthwhile to identify additional plant-derived chemicals that impact *Agrobacterium* pathogenicity and rhizospheric fitness. In addition, it will be very interesting to elucidate *Agrobacterium* signaling pathways and underlying regulatory mechanisms responsible for the precise perception and response to these plant-derived signals, especially at the early stage of *Agrobacterium*–plant interaction.

## Conflict of Interest Statement

The authors declare that the research was conducted in the absence of any commercial or financial relationships that could be construed as a potential conflict of interest.

## ABBREVIATIONS

AS: acetosyringone
*chv* genes: chromosome encoded virulence genes
CK: cytokinin
DIMBOA: 2,4-dihydroxy-7-methoxy-2H-1,4-benzixazin-3(4H)-one
ET: ethylene
GABA: γ-amino butyric acid
IAA: indole acetic acid
MDIBOA: 2-hydroxy-4,7-dimethoxybenzoxazin-3-one
QS: quorum sensing
SA: salicylic acid
T3SS: type III secretion system
T4SS: type IV secretion system
T6SS: type VI secretion system
T-DNA: transferred DNA
Ti-plasmid: tumor-inducing plasmid
*vir* genes: virulence genes

## References

[B1] Anand A. Uppalapati S. R. Ryu C. M. Allen S. N. Kang L. Tang Y. (2008). Salicylic acid and systemic acquired resistance play a role in attenuating crown gall disease caused by *Agrobacterium tumefaciens*. *Plant Physiol.* 146 703–715 10.1104/pp.107.111302 18156296 PMC2245820

[B2] Badri D. V. Vivanco J. M. (2009). Regulation and function of root exudates. *Plant Cell Environ.* 32 666–681 10.1111/j.1365-3040.2008.01926.x 19143988

[B3] Bais H. P. Prithiviraj B. Jha A. K. Ausubel F. M. Vivanco J. M. (2005). Mediation of pathogen resistance by exudation of antimicrobials from roots. *Nature* 434 217–221 10.1038/nature03356 15759001

[B4] Banta L. M. Joerger R. D. Howitz V. R. Campbell A. M. Binns A. N. (1994). Glu-255 outside the predicted ChvE binding site in VirA is crucial for sugar enhancement of acetosyringone perception by *Agrobacterium tumefaciens*. *J. Bacteriol.* 176 3242–3249 8195079 10.1128/jb.176.11.3242-3249.1994 PMC205494

[B5] Beck von Bodman S. Hayman G. T. Farrand S. K. (1992). Opine catabolism and conjugal transfer of the nopaline Ti plasmid pTiC58 are coordinately regulated by a single repressor. *Proc. Natl. Acad. Sci. U.S.A.* 89 643–647 10.1073/pnas.89.2.643 1731335 PMC48295

[B6] Brencic A. Eberhard A. Winans S. C. (2004). Signal quenching, detoxification and mineralization of vir gene-inducing phenolics by the VirH2 protein of *Agrobacterium tumefaciens*. *Mol. Microbiol.* 51 1103–1115 10.1046/j.1365-2958.2003.03887.x 14763983

[B7] Brencic A. Winans S. C. (2005). Detection of and response to signals involved in host–microbe interactions by plant-associated bacteria. *Microbiol. Mol. Biol. Rev.* 69 155–194 10.1128/MMBR.69.1.155-194.2005 15755957 PMC1082791

[B8] Brugiere N. Dubois F. Limami A. M. Lelandais M. Roux Y. Sangwan R. S. (1999). Glutamine synthetase in the phloem plays a major role in controlling proline production. *Plant Cell* 11 1995–2012 10.1105/tpc.11.10.1995 10521528 PMC144111

[B9] Buttner D. He S. Y. (2009). The T3SS, type III protein secretion in plant pathogenic bacteria. *Plant Physiol.* 150 1656–1664 10.1104/pp.109.139089 19458111 PMC2719110

[B10] Cangelosi G. A. Ankenbauer R. G. Nester E. W. (1990). Sugars induce the *Agrobacterium virulence* genes through a periplasmic binding protein and a transmembrane signal protein. *Proc. Natl. Acad. Sci. U.S.A.* 87 6708–6712 10.1073/pnas.87.17.6708 2118656 PMC54606

[B11] Cangelosi G. A. Martinetti G. Leigh J. A. Lee C. C. Thienes C. Nester E. W. (1989). Role for *Agrobacterium tumefaciens* ChvA protein in export of beta-1,2-glucan. *J. Bacteriol.* 171 1609–1615 2921245 10.1128/jb.171.3.1609-1615.1989 PMC209788

[B12] Carlier A. Chevrot R. Dessaux Y. Faure D. (2004). The assimilation of gamma-butyrolactone in *Agrobacterium tumefaciens* C58 interferes with the accumulation of the N-acyl-homoserine lactone signal. *Mol. Plant Microbe Interact.* 17 951–957 10.1094/MPMI.2004.17.9.951 15384485

[B13] Cascales E. Atmakuri K. Sarkar M. K. Christie P. J. (2013). DNA substrate-induced activation of the *Agrobacterium* VirB/VirD4 type IV secretion system. *J. Bacteriol.* 195 2691–2704 10.1128/JB.00114-13 23564169 PMC3676061

[B14] Cascales E. Christie P. J. (2003). The versatile bacterial type IV secretion systems. *Nat. Rev. Microbiol.* 1 137–149 10.1038/nrmicro753 15035043 PMC3873781

[B15] Chai Y. Tsai C. S. Cho H. Winans S. C. (2007). Reconstitution of the biochemical activities of the AttJ repressor and the AttK, AttL, and AttM catabolic enzymes of *Agrobacterium tumefaciens*. *J. Bacteriol.* 189 3674–3679 10.1128/JB.01274-06 17307843 PMC1855881

[B16] Chang C. Shockey J. A. (1999). The ethylene-response pathway: signal perception to gene regulation. *Curr. Opin. Plant Biol.* 2 352–358 10.1016/S1369-5266(99)00004-7 10508761

[B17] Chang C. H. Winans S. C. (1992). Functional roles assigned to the periplasmic, linker, and receiver domains of the *Agrobacterium tumefaciens* VirA protein. *J. Bacteriol.* 174 7033–7039 1400253 10.1128/jb.174.21.7033-7039.1992 PMC207384

[B18] Charles T. C. Nester E. W. (1993). A chromosomally encoded two-component sensory transduction system is required for virulence of *Agrobacterium tumefaciens*. *J. Bacteriol.* 175 6614–6625 8407839 10.1128/jb.175.20.6614-6625.1993 PMC206773

[B19] Chen C. Y. Winans S. C. (1991). Controlled expression of the transcriptional activator gene virG in *Agrobacterium tumefaciens* by using the *Escherichia coli* lac promoter. *J. Bacteriol.* 173 1139–1144 1991713 10.1128/jb.173.3.1139-1144.1991 PMC207234

[B20] Chevrot R. Rosen R. Haudecoeur E. Cirou A. Shelp B. J. Ron E. (2006). GABA controls the level of quorum-sensing signal in *Agrobacterium tumefaciens*. *Proc. Natl. Acad. Sci. U.S.A.* 103 7460–7464 10.1073/pnas.0600313103 16645034 PMC1464361

[B21] Cho H. Winans S. C. (2005). VirA and VirG activate the Ti plasmid repABC operon, elevating plasmid copy number in response to wound-released chemical signals. *Proc. Natl. Acad. Sci. U.S.A.* 102 14843–14848 10.1073/pnas.0503458102 16195384 PMC1253548

[B22] Christie P. J. (2004). Type IV secretion: the *Agrobacterium* VirB/D4 and related conjugation systems. *Biochim. Biophys. Acta* 1694 219–234 Review. 10.1016/j.bbamcr.2004.02.013 15546668 PMC4845649

[B23] Citovsky V. Kozlovsky S. V. Lacroix B. Zaltsman A. Dafny-Yelin M. Vyas S. (2007). Biological systems of the host cell involved in *Agrobacterium* infection. *Cell Microbiol.* 9 9–20 10.1111/j.1462-5822.2006.00830.x 17222189

[B24] Citovsky V. Zupan J. Warnick D. Zambryski P. (1992). Nuclear localization of *Agrobacterium* VirE2 protein in plant cells. *Science* 256 1802–1805 10.1126/science.1615325 1615325

[B25] Deeken R. Engelmann J. C. Efetova M. Czirjak T. Müller T. Kaiser W. M. (2006). An integrated view of gene expression and solute profiles of *Arabidopsis* tumors: a genome-wide approach. *Plant Cell* 18 3617–3634 10.1105/tpc.106.044743 17172353 PMC1785400

[B26] Dessaux Y. Petit A. Farrand S. K. Murphy P. J. (1998). “Opines and opine-like molecules involved in plant–Rhizobiaceae interactions,” in *The Rhizobiaceae: Molecular Biology of Model Plant-associated Bacteria* eds Spaink H. P. Kondorosi A. Hooykaas P. J. J. (Dordrecht: Kluwer Academic Publishers), 173–197

[B27] Dixon R. A. Paiva N. L. (1995). Stress-induced phenylpropanoid metabolism. *Plant Cell* 7 1085–1097 10.1105/tpc.7.7.1085 12242399 PMC160915

[B28] Djamei A. Pitzschke A. Nakagami H. Rajh I. Hirt H. (2007). Trojan horse strategy in *Agrobacterium* transformation: abusing MAPK defense signaling. *Science* 318 453–456 10.1126/science.1148110 17947581

[B29] Doty S. L. Chang M. Nester E. W. (1993). The chromosomal virulence gene, chvE, of *Agrobacterium tumefaciens* is regulated by a LysR family member. *J. Bacteriol.* 175 7880–7886 8253677 10.1128/jb.175.24.7880-7886.1993 PMC206966

[B30] Fuqua C. Winans S. C. (1996a). Conserved cis-acting promoter elements are required for density-dependent transcription of *Agrobacterium tumefaciens* conjugal transfer genes. *J. Bacteriol.* 178 435–440 8550463 10.1128/jb.178.2.435-440.1996 PMC177675

[B31] Fuqua C. Winans S. C. (1996b). Localization of OccR-activated and TraR-activated promoters that express two ABC-type permeases and the traR gene of Ti plasmid pTiR10. *Mol. Microbiol.* 20 1199–1210 10.1111/j.1365-2958.1996.tb02640.x 8809772

[B32] Fuqua W. C. Winans S. C. (1994). A LuxR-LuxI type regulatory system activates *Agrobacterium* Ti plasmid conjugal transfer in the presence of a plant tumor metabolite. *J. Bacteriol.* 176 2796–2806 8188582 10.1128/jb.176.10.2796-2806.1994 PMC205432

[B33] Gao R. Lynn D. G. (2005). Environmental pH sensing: resolving the VirA/VirG two-component system inputs for *Agrobacterium* pathogenesis. *J. Bacteriol.* 187 2182–2189 10.1128/JB.187.6.2182-2189.2005 15743967 PMC1064044

[B34] Gelvin S. B. (2003). *Agrobacterium*-mediated plant transformation: the biology behind the “Gene-Jockeying” tool. *Microbiol.* *Mol. Biol. Rev* 67 16–37 10.1128/MMBR.67.1.16-37.2003 PMC150518 12626681

[B35] Gelvin S. B. (2012). Traversing the cell: *Agrobacterium* T-DNA’s journey to the host genome. *Front. Plant Sci.* 3:52 10.3389/fpls.2012.00052 PMC3355731 22645590

[B36] Haudecoeur E. Planamente S. Cirou A. Tannieres M. Shelp B. J. Morera S. (2009). Proline antagonizes GABA-induced quenching of quorum-sensing in *Agrobacterium tumefaciens*. *Proc. Natl. Acad. Sci. U.S.A.* 106 14587–14592 10.1073/pnas.0808005106 19706545 PMC2732848

[B37] He F. Nair G. R. Soto C. S. Chang Y. Hsu L. Ronzone E. (2009). Molecular basis of ChvE function in sugar binding, sugar utilization, and virulence in *Agrobacterium tumefaciens*. *J. Bacteriol.* 191 5802–5813 10.1128/JB.00451-09 19633083 PMC2737963

[B38] Heath J. D. Boulton M. I. Raineri D. M. Doty S. L. Mushegian A. R. Charles T. C. (1997). Discrete regions of the sensor protein virA determine the strain-specific ability of *Agrobacterium* to agroinfect maize. *Mol. Plant Microbe Interact.* 10 221–227 10.1094/MPMI.1997.10.2.221 9057328

[B39] Hess K. M. Dudley M. W. Lynn D. G. Joerger R. D. Binns A. N. (1991). Mechanism of phenolic activation of *Agrobacterium virulence* genes: development of a specific inhibitor of bacterial sensor/response systems. *Proc. Natl. Acad. Sci. U.S.A.* 88 7854–7858 10.1073/pnas.88.17.7854 1909032 PMC52402

[B40] Hong S. B. Dessaux Y. Chilton W. S. Farrand S. K. (1993). Organization and regulation of the mannopine cyclase-associated opine catabolism genes in *Agrobacterium tumefaciens* 15955. *J. Bacteriol.* 175 401–410 8380402 10.1128/jb.175.2.401-410.1993 PMC196154

[B41] Hu X. Zhao J. Degrado W. F. Binns A. N. (2013). *Agrobacterium tumefaciens* recognizes its host environment using ChvE to bind diverse plant sugars as virulence signals. *Proc. Natl. Acad. Sci. U.S.A.* 110 678–683 10.1073/pnas.1215033110 23267119 PMC3545744

[B42] Huckelhoven R. (2007). Transport and secretion in plant-microbe interactions. *Curr. Opin. Plant Biol.* 10 573–579 10.1016/j.pbi.2007.08.002 17875397

[B43] Hwang I. Li P. L. Zhang L. Piper K. R. Cook D. M. Tate M. E. (1994). TraI, a LuxI homologue, is responsible for production of conjugation factor, the Ti plasmid N-acylhomoserine lactone autoinducer. *Proc. Natl. Acad. Sci. U.S.A.* 91 4639–4643 10.1073/pnas.91.11.4639 8197112 PMC43843

[B44] Hwang I. Smyth A. J. Luo Z. Q. Farrand S. K. (1999). Modulating quorum sensing by antiactivation: TraM interacts with TraR to inhibit activation of Ti plasmid conjugal transfer genes. *Mol. Microbiol.* 34 282–294 10.1046/j.1365-2958.1999.01595.x 10564472

[B45] Jia Y. H. Li L. P. Hou Q. M. Pan S. Q. (2002). An *Agrobacterium* gene involved in tumorigenesis encodes an outer membrane protein exposed on the bacterial cell surface. *Gene* 284 113–124 10.1016/S0378-1119(02)00385-2 11891052

[B46] Jin S. Roitsch T. Ankenbauer R. G. Gordon M. P. Nester E. W. (1990a). The VirA protein of *Agrobacterium tumefaciens* is autophosphorylated and is essential for vir gene regulation. *J. Bacteriol.* 172 525–530 2404940 10.1128/jb.172.2.525-530.1990 PMC208473

[B47] Jin S. G. Prusti R. K. Roitsch T. Ankenbauer R. G. Nester E. W. (1990b). Phosphorylation of the VirG protein of *Agrobacterium tumefaciens* by the autophosphorylated VirA protein: essential role in biological activity of VirG. *J. Bacteriol.* 172 4945–4950 2394678 10.1128/jb.172.9.4945-4950.1990 PMC213149

[B48] Jin S. G. Roitsch T. Christie P. J. Nester E. W. (1990c). The regulatory VirG protein specifically binds to a cis-acting regulatory sequence involved in transcriptional activation of *Agrobacterium tumefaciens* virulence genes. *J. Bacteriol.* 172 531–537 2404941 10.1128/jb.172.2.531-537.1990 PMC208474

[B49] Kalogeraki V. S. Winans S. C. (1998). Wound-released chemical signals may elicit multiple responses from an *Agrobacterium tumefaciens* strain containing an octopine-type Ti plasmid. *J. Bacteriol.* 180 5660–5667 9791116 10.1128/jb.180.21.5660-5667.1998 PMC107625

[B50] Kalogeraki V. S. Zhu J. Stryker J. L. Winans S. C. (2000). The right end of the vir region of an octopine-type Ti plasmid contains four new members of the vir regulon that are not essential for pathogenesis. *J. Bacteriol.* 182 1774–1778 10.1128/JB.182.6.1774-1778.2000 10692388 PMC94480

[B51] Kim H. Farrand S. K. (1997). Characterization of the acc operon from the nopaline-type Ti plasmid pTiC58, which encodes utilization of agrocinopines A and B and susceptibility to agrocin 84. *J. Bacteriol.* 179 7559–7572 9393724 10.1128/jb.179.23.7559-7572.1997 PMC179710

[B52] Kim H. S. Yi H. Myung J. Piper K. R. Farrand S. K. (2008). Opine-based *Agrobacterium* competitiveness: dual expression control of the agrocinopine catabolism (acc) operon by agrocinopines and phosphate levels. *J. Bacteriol.* 190 3700–3711 10.1128/JB.00067-08 18344359 PMC2395003

[B53] Korbei B. Luschnig C. (2011). Cell polarity: PIN it down! *Curr. Biol.* 21 R197–R199 10.1016/j.cub.2011.01.062 21377099

[B54] Lacroix B. Loyter A. Citovsky V. (2008). Association of the *Agrobacterium* T-DNA-protein complex with plant nucleosomes. *Proc. Natl. Acad. Sci. U.S.A.* 105 15429–15434 10.1073/pnas.0805641105 18832163 PMC2563108

[B55] Lai E. M. Shih H. W. Wen S. R. Cheng M. W. Hwang H. H. Chiu S. H. (2006). Proteomic analysis of *Agrobacterium tumefaciens* response to the Vir gene inducer acetosyringone. *Proteomics* 6 4130–4136 10.1002/pmic.200600254 16791832

[B56] Lee C. W. Efetova M. Engelmann J. C. Kramell R. Wasternack C. Ludwig-Muller J. (2009). *Agrobacterium tumefaciens* promotes tumor induction by modulating pathogen defense in *Arabidopsis thaliana*. *Plant Cell* 21 2948–2962 10.1105/tpc.108.064576 19794116 PMC2768927

[B57] Lee K. Dudley M. W. Hess K. M. Lynn D. G. Joerger R. D. Binns A. N. (1992). Mechanism of activation of *Agrobacterium virulence* genes: identification of phenol-binding proteins. *Proc. Natl. Acad. Sci. U.S.A.* 89 8666–8670 10.1073/pnas.89.18.8666 1528878 PMC49981

[B58] Lee Y. W. Jin S. Sim W. S. Nester E. W. (1995). Genetic evidence for direct sensing of phenolic compounds by the VirA protein of *Agrobacterium tumefaciens*. *Proc. Natl. Acad. Sci. U.S.A.* 92 12245–12249 10.1073/pnas.92.26.12245 8618878 PMC40333

[B59] Leroux B. Yanofsky M. F. Winans S. C. Ward J. E. Ziegler S. F. Nester E. W. (1987). Characterization of the virA locus of *Agrobacterium tumefaciens*: a transcriptional regulator and host range determinant. *EMBO J.* 6 849–856 3595559 10.1002/j.1460-2075.1987.tb04830.x PMC553474

[B60] Li J. Krichevsky A. Vaidya M. Tzfira T. Citovsky V. (2005). Uncoupling of the functions of the *Arabidopsis* VIP1 protein in transient and stable plant genetic transformation by *Agrobacterium*. *Proc. Natl. Acad. Sci. U.S.A.* 102 5733–5738 10.1073/pnas.0404118102 15824315 PMC556277

[B61] Li L. Jia Y. Hou Q. Charles T. C. Nester E. W. Pan S. Q. (2002). A global pH sensor: *Agrobacterium* sensor protein ChvG regulates acid-inducible genes on its two chromosomes and Ti plasmid. *Proc. Natl. Acad. Sci. U.S.A.* 99 12369–12374 10.1073/pnas.192439499 12218184 PMC129451

[B62] Li P. L. Farrand S. K. (2000). The replicator of the nopaline-type Ti plasmid pTiC58 is a member of the repABC family and is influenced by the TraR-dependent quorum-sensing regulatory system. *J. Bacteriol.* 182 179–188 10.1128/JB.182.1.179-188.2000 10613878 PMC94255

[B63] Liu C. N. Li X. Q. Gelvin S. B. (1992). Multiple copies of virG enhance the transient transformation of celery, carrot and rice tissues by *Agrobacterium tumefaciens*. *Plant Mol. Biol.* 20 1071–1087 10.1007/BF00028894 1463842

[B64] Liu P. Nester E. W. (2006). Indoleacetic acid, a product of transferred DNA, inhibits vir gene expression and growth of *Agrobacterium tumefaciens* C58. *Proc. Natl. Acad. Sci. U.S.A.* 103 4658–4662 10.1073/pnas.0600366103 16537403 PMC1450227

[B65] Liu P. Wood D. Nester E. W. (2005). Phosphoenolpyruvate carboxykinase is an acid-induced, chromosomally encoded virulence factor in *Agrobacterium tumefaciens*. *J. Bacteriol.* 187 6039–6045 10.1128/JB.187.17.6039-6045.2005 16109945 PMC1196135

[B66] Magori S. Citovsky V. (2011). Agrobacterium counteracts host-induced degradation of its effector F-box protein. *Sci. Signal.* 4:ra69 10.1126/scisignal.2002124 PMC9472992 22009152

[B67] Mantis N. J. Winans S. C. (1993). The chromosomal response regulatory gene chvI of *Agrobacterium tumefaciens* complements an *Escherichia coli* phoB mutation and is required for virulence. *J. Bacteriol.* 175 6626–6636 8407840 10.1128/jb.175.20.6626-6636.1993 PMC206774

[B68] Mathesius U. Mulders S. Gao M. Teplitski M. Caetano-Anolles G. Rolfe B. G. (2003). Extensive and specific responses of a eukaryote to bacterial quorum-sensing signals. *Proc. Natl. Acad. Sci. U.S.A.* 100 1444–1449 10.1073/pnas.262672599 12511600 PMC298792

[B69] McCullen C. A. Binns A. N. (2006). *Agrobacterium tumefaciens* and plant cell interactions and activities required for interkingdom macromolecular transfer. *Annu. Rev. Cell Dev. Biol.* 22 101–127 10.1146/annurev.cellbio.22.011105.102022 16709150

[B70] Melchers L. S. Regensburg-Tuink A. J. Schilperoort R. A. Hooykaas P. J. (1989). Specificity of signal molecules in the activation of *Agrobacterium virulence* gene expression. *Mol. Microbiol.* 3 969–977 10.1111/j.1365-2958.1989.tb00246.x 2796734

[B71] Morel P. Powell B. S. Kado C. I. (1990). Demonstration of 3 functional domains responsible for a kinase activity in VirA, a transmembrane sensory protein encoded by the Ti plasmid of *Agrobacterium tumefaciens*. *C. R. Acad. Sci. III* 310 21–26 2105145

[B72] Nair G. R. Lai X. Wise A. A. Rhee B. W. Jacobs M. Binns A. N. (2011). The integrity of the periplasmic domain of the VirA sensor kinase is critical for optimal coordination of the virulence signal response in *Agrobacterium tumefaciens*. *J. Bacteriol.* 193 1436–1448 10.1128/JB.01227-10 21216996 PMC3067612

[B73] Nam J. Matthysse A. G. Gelvin S. B. (1997). Differences in susceptibility of *Arabidopsis* ecotypes to crown gall disease may result from a deficiency in T-DNA integration. *Plant Cell* 9 317–333 10.1105/tpc.9.3.317 9090878 PMC156921

[B74] Nautiyal C. S. Dion P. (1990). Characterization of the opine-utilizing microflora associated with samples of soil and plants. *Appl. Environ. Microbiol.* 56 2576–2579 16348265 10.1128/aem.56.8.2576-2579.1990 PMC184770

[B75] Nonaka S. Yuhashi K. Takada K. Sugaware M. Minamisawa K. Ezura H. (2008). Ethylene production in plants during transformation suppresses vir gene expression in *Agrobacterium tumefaciens*. *New Phytol.* 178 647–656 10.1111/j.1469-8137.2008.02400.x 18331427

[B76] Pappas K. M. Winans S. C. (2003). A LuxR-type regulator from *Agrobacterium tumefaciens* elevates Ti plasmid copy number by activating transcription of plasmid replication genes. *Mol. Microbiol.* 48 1059–1073 10.1046/j.1365-2958.2003.03488.x 12753196

[B77] Parke D. Ornston L. N. Nester E. W. (1987). Chemotaxis to plant phenolic inducers of virulence genes is constitutively expressed in the absence of the Ti plasmid in *Agrobacterium tumefaciens*. *J. Bacteriol.* 169 5336–5338 3667536 10.1128/jb.169.11.5336-5338.1987 PMC213951

[B78] Pazour G. J. Das A. (1990). Characterization of the VirG binding site of *Agrobacterium tumefaciens*. *Nucleic Acids Res.* 18 6909–6913 10.1093/nar/18.23.6909 2263453 PMC332749

[B79] Peng W. T. Lee Y. W. Nester E. W. (1998). The phenolic recognition profiles of the *Agrobacterium tumefaciens* VirA protein are broadened by a high level of the sugar binding protein ChvE. *J. Bacteriol.* 180 5632–5638 9791112 10.1128/jb.180.21.5632-5638.1998 PMC107621

[B80] Phillips D. A. Fox T. C. King M. D. Bhuvaneswari T. V. Teuber L. R. (2004). Microbial products trigger amino acid exudation from plant roots. *Plant Physiol.* 136 2887–2894 10.1104/pp.104.044222 15347793 PMC523350

[B81] Pinto U. M. Pappas K. M. Winans S. C. (2012). The ABCs of plasmid replication and segregation. *Nat. Rev. Microbiol.* 10 755–765 10.1038/nrmicro2882 23070556

[B82] Piper K. R. Beck Von Bodman S. Farrand S. K. (1993). Conjugation factor of *Agrobacterium tumefaciens* regulates Ti plasmid transfer by autoinduction. *Nature* 362 448–450 10.1038/362448a0 8464476

[B83] Piper K. R. Beck Von Bodman S. Hwang I. Farrand S. K. (1999). Hierarchical gene regulatory systems arising from fortuitous gene associations: controlling quorum sensing by the opine regulon in *Agrobacterium*. *Mol. Microbiol.* 32 1077–1089 10.1046/j.1365-2958.1999.01422.x 10361309

[B84] Pitzschke A. (2013). Infection and plant defense-transformation success hangs by a thread. *Front. Plant Sci.* 4:519 10.3389/fpls.2013.00519 PMC3866890 24391655

[B85] Ritchie A. J. Jansson A. Stallberg J. Nilsson P. Lysaght P. Cooley M. A. (2005). The *Pseudomonas aeruginosa* quorum-sensing molecule N-3-(oxododecanoyl)-L-homoserine lactone inhibits T-cell differentiation and cytokine production by a mechanism involving an early step in T-cell activation. *Infect. Immun.* 73 1648–1655 10.1128/IAI.73.3.1648-1655.2005 15731065 PMC1064928

[B86] Rivoal J. Hanson A. D. (1994). Metabolic control of anaerobic glycolysis (overexpression of lactate dehydrogenase in transgenic tomato roots supports the Davies–Roberts hypothesis and points to a critical role for lactate secretion. *Plant Physiol.* 106 1179–1185 12232401 10.1104/pp.106.3.1179 PMC159647

[B87] Rogowsky P. M. Close T. J. Chimera J. A. Shaw J. J. Kado C. I. (1987). Regulation of the vir genes of *Agrobacterium tumefaciens* plasmid pTiC58. *J. Bacteriol.* 169 5101–5112 2822665 10.1128/jb.169.11.5101-5112.1987 PMC213914

[B88] Roitsch T. Wang H. Jin S. G. Nester E. W. (1990). Mutational analysis of the VirG protein, a transcriptional activator of *Agrobacterium tumefaciens* virulence genes. *J. Bacteriol.* 172 6054–6060 2211523 10.1128/jb.172.10.6054-6060.1990 PMC526929

[B89] Sahi S. V. Chilton M. D. Chilton W. S. (1990). Corn metabolites affect growth and virulence of *Agrobacterium tumefaciens*. *Proc. Natl. Acad. Sci. U.S.A.* 87 3879–3883 10.1073/pnas.87.10.3879 11607078 PMC54007

[B90] Savka M. A. Farrand S. K. (1992). Mannityl opine accumulation and exudation by transgenic tobacco. *Plant Physiol.* 98 784–789 10.1104/pp.98.2.784 16668714 PMC1080263

[B91] Shaw C. H. Ashby A. M. Brown A. Royal C. Loake G. J. (1988). virA and virG are the Ti-plasmid functions required for chemotaxis of *Agrobacterium tumefaciens* towards acetosyringone. *Mol. Microbiol.* 2 413–417 10.1111/j.1365-2958.1988.tb00046.x 3398775

[B92] Shimoda N. Toyoda-Yamamoto A. Aoki S. Machida Y. (1993). Genetic evidence for an interaction between the VirA sensor protein and the ChvE sugar-binding protein of *Agrobacterium*. *J. Biol. Chem.* 268 26552–26558 8253785

[B93] Stachel S. E. Messens M. Van Montagu A. Zambryski P. (1985). Identification of the signal molecules produced by wounded plant cells that activate T-DNA transfer in *Agrobacterium tumefaciens*. *Nature* 318 624–629 10.1038/318624a0

[B94] Stachel S. E. Nester E. W. (1986). The genetic and transcriptional organization of the vir region of the A6 Ti plasmid of *Agrobacterium tumefaciens*. *EMBO J.* 5 1445–1454 3017694 10.1002/j.1460-2075.1986.tb04381.x PMC1166964

[B95] Stachel S. E. Nester E. W. Zambryski P. C. (1986). A plant cell factor induces *Agrobacterium tumefaciens* vir gene expression. *Proc. Natl. Acad. Sci. U.S.A.* 83 379–383 10.1073/pnas.83.2.379 16593648 PMC322862

[B96] Thomashow M. F. Hugly S. Buchholz W. G. Thomashow L. S. (1986). Molecular basis for the auxin-independent phenotype of crown gall tumor tissues. *Science* 231 616–618 10.1126/science.3511528 3511528

[B97] Toyoda-Yamamoto A. Shimoda N. Machida Y. (2000). Genetic analysis of the signal-sensing region of the histidine protein kinase VirA of *Agrobacterium tumefaciens*. *Mol. Gen. Genet.* 263 939–947 10.1007/PL00008694 10954079

[B98] Turk S. C. Van Lange R. P. Regensburg-Tuink T. J. Hooykaas P. J. (1994). Localization of the VirA domain involved in acetosyringone-mediated vir gene induction in *Agrobacterium tumefaciens*. *Plant Mol. Biol.* 25 899–907 10.1007/BF00028884 8075405

[B99] Tzfira T. Vaidya M. Citovsky V. (2001). VIP1 an *Arabidopsis* protein that interacts with *Agrobacterium* VirE2, is involved in VirE2 nuclear import and *Agrobacterium* infectivity. *EMBO J.* 20 3596–3607 10.1093/emboj/20.13.3596 11432846 PMC125502

[B100] Vergunst A. C. Schrammeijer B. Den Dulk-Ras A. De Vlaam C. M. Regensburg-Tuink T. J. Hooykaas P. J. (2000). VirB/D4-dependent protein translocation from *Agrobacterium* into plant cells. *Science* 290 979–982 10.1126/science.290.5493.979 11062129

[B101] Veselov D. Langhans M. Hartung W. Aloni R. Feussner I. Götz C. (2003). Development of *Agrobacterium tumefaciens* C58-induced plant tumors, and impact on host shoots are controlled by a cascade of jasmonic acid, auxin, cytokinin, ethylene, and abscisic acid. *Planta* 216 512–522 12520344 10.1007/s00425-002-0883-5

[B102] Wachter R. Langhans M. Aloni R. Gotz S. Weilmunster A. Koops A. (2003). Vascularization, high-volume solution flow, and localized roles for enzymes of sucrose metabolism during tumorigenesis by *Agrobacterium tumefaciens*. *Plant Physiol.* 133 1024–1037 10.1104/pp.103.028142 14526106 PMC281599

[B103] Wagner C. Zimmermann S. Brenner-Weiss G. Hug F. Prior B. Obst U. (2007). The quorum-sensing molecule N-3-oxododecanoyl homoserine lactone (3OC12-HSL) enhances the host defence by activating human polymorphonuclear neutrophils (PMN). *Anal. Bioanal. Chem.* 387 481–487 10.1007/s00216-006-0698-695 16906383

[B104] Walker T. S. Bais H. P. Grotewold E. Vivanco J. M. (2003). Root exudation and rhizosphere biology. *Plant Physiol.* 132 44–51 10.1104/pp.102.019661 12746510 PMC1540314

[B105] Wang F. F. Cui X. K. Sun Y. Dong C. H. (2013). Ethylene signaling and regulation in plant growth and stress responses. *Plant Cell Rep.* 32 1099–1109 10.1007/s00299-013-1421-6 23525746

[B106] Wang K. Herrera-Estrella A. Van Montagu M. (1990). Overexpression of virD1 and virD2 genes in *Agrobacterium tumefaciens* enhances T-complex formation and plant transformation. *J. Bacteriol.* 172 4432–4440 2165478 10.1128/jb.172.8.4432-4440.1990 PMC213272

[B107] Wang L. Helmann J. D. Winans S. C. (1992). The *A. tumefaciens* transcriptional activator OccR causes a bend at a target promoter, which is partially relaxed by a plant tumor metabolite. *Cell* 69 659–667 10.1016/0092-8674(92)90229-6 1586946

[B108] Wang P. Bi S. Ma L. Han W. (2006). Aluminum tolerance of two wheat cultivars (Brevor and Atlas66) in relation to their rhizosphere pH and organic acids exuded from roots. *J. Agric. Food Chem.* 54 10033–10039 10.1021/jf0611769 17177538

[B109] White C. E. Winans S. C. (2007). Cell–cell communication in the plant pathogen *Agrobacterium tumefaciens*. *Philos. Trans. R. Soc. Lond. B Biol. Sci.* 362 1135–1148 10.1098/rstb.2007.2040 17360279 PMC2435578

[B110] Wilms I. Moller P. Stock A. M. Gurski R. Lai E. M. Narberhaus F. (2012). Hfq influences multiple transport systems and virulence in the plant pathogen *Agrobacterium tumefaciens*. *J. Bacteriol.* 194 5209–5217 10.1128/JB.00510-12 22821981 PMC3457239

[B111] Wilms I. Voss B. Hess W. R. Leichert L. I. Narberhaus F. (2011). Small RNA-mediated control of the *Agrobacterium tumefaciens* GABA binding protein. *Mol. Microbiol.* 80 492–506 10.1111/j.1365-2958.2011.07589.x 21320185

[B112] Winans S. C. (1990). Transcriptional induction of an *Agrobacterium* regulatory gene at tandem promoters by plant-released phenolic compounds, phosphate starvation, and acidic growth media. *J. Bacteriol.* 172 2433–2438 2185220 10.1128/jb.172.5.2433-2438.1990 PMC208880

[B113] Winans S. C. (1992). Two-way chemical signaling in *Agrobacterium*–plant interactions. *Microbiol. Rev.* 56 12–31 1579105 10.1128/mr.56.1.12-31.1992 PMC372852

[B114] Winans S. C. Ebert P. R. Stachel S. E. Gordon M. P. Nester E. W. (1986). A gene essential for *Agrobacterium virulence* is homologous to a family of positive regulatory loci. *Proc. Natl. Acad. Sci. U.S.A.* 83 8278–8282 10.1073/pnas.83.21.8278 3022288 PMC386911

[B115] Wise A. A. Fang F. Lin Y. H. He F. Lynn D. G. Binns A. N. (2010). The receiver domain of hybrid histidine kinase VirA: an enhancing factor for vir gene expression in *Agrobacterium tumefaciens*. *J. Bacteriol.* 192 1534–1542 10.1128/JB.01007-09 20081031 PMC2832513

[B116] Wright E. L. Deakin W. J. Shaw C. H. (1998). A chemotaxis cluster from *Agrobacterium tumefaciens*. *Gene* 220 83–89 10.1016/S0378-1119(98)00438-7 9767126

[B117] Wu C. F. Lin J. S. Shaw G. C. Lai E. M. (2012). Acid-induced type VI secretion system is regulated by ExoR-ChvG/ChvI signaling cascade in *Agrobacterium tumefaciens*. *PLoS Pathog.* 8:e1002938 10.1371/journal.ppat.1002938 PMC3460628 23028331

[B118] Xia J. H. Roberts J. (1994). Improved cytoplasmic pH regulation, increased lactate eﬄux, and reduced cytoplasmic lactate levels are biochemical traits expressed in root tips of whole maize seedlings acclimated to a low-oxygen environment. *Plant Physiol.* 105 651–657 12232232 10.1104/pp.105.2.651 PMC159406

[B119] Yanofsky M. F. Porter S. G. Young C. Albright L. M. Gordon M. P. Nester E. W. (1986). The virD operon of *Agrobacterium tumefaciens* encodes a site-specific endonuclease. *Cell* 47 471–477 10.1016/0092-8674(86)90604-5 3021341

[B120] Yuan Z. C. Edlind M. P. Liu P. Saenkham P. Banta L. M. Wise A. A. (2007). The plant signal salicylic acid shuts down expression of the vir regulon and activates quormone-quenching genes in *Agrobacterium*. *Proc. Natl. Acad. Sci. U.S.A.* 104 11790–11795 10.1073/pnas.0704866104 17606909 PMC1905925

[B121] Yuan Z. C. Haudecoeur E. Faure D. Kerr K. F. Nester E. W. (2008a). Comparative transcriptome analysis of *Agrobacterium tumefaciens* in response to plant signal salicylic acid, indole-3-acetic acid and gamma-amino butyric acid reveals signalling cross-talk and *Agrobacterium*–plant co-evolution. *Cell Microbiol.* 10 2339–2354 10.1111/j.1462-5822.2008.01215.x 18671824

[B122] Yuan Z. C. Liu P. Saenkham P. Kerr K. Nester E. W. (2008b). Transcriptome profiling and functional analysis of *Agrobacterium tumefaciens* reveals a general conserved response to acidic conditions (pH 5.5) and a complex acid-mediated signaling involved in *Agrobacterium*–plant interactions. *J. Bacteriol.* 190 494–507 10.1128/JB.01387-1387 17993523 PMC2223696

[B123] Yuan Z. C. Williams M. (2012). A really useful pathogen, *Agrobacterium tumefaciens*. *Plant Cell* 24:tpc.112.tt1012. 10.1105/tpc.112.tt1012 PMC3517252 23213133

[B124] Zhang J. Boone L. Kocz R. Zhang C. Binns A. N. Lynn D. G. (2000). At the maize/*Agrobacterium interface*: natural factors limiting host transformation. *Chem. Biol.* 7 611–621 10.1016/S1074-5521(00)00007-7 11048952

[B125] Zhang L. Murphy P. J. Kerr A. Tate M. E. (1993). *Agrobacterium* conjugation and gene regulation by N-acyl-L-homoserine lactones. *Nature* 362 446–448 10.1038/362446a0 8464475

[B126] Zhu J. Oger P. M. Schrammeijer B. Hooykaas P. J. Farrand S. K. Winans S. C. (2000). The bases of crown gall tumorigenesis. *J. Bacteriol.* 182 3885–3895 10.1128/JB.182.14.3885-3895.2000 10869063 PMC94570

[B127] Zhu J. Winans S. C. (1999). Autoinducer binding by the quorum-sensing regulator TraR increases affinity for target promoters in vitro and decreases TraR turnover rates in whole cells. *Proc. Natl. Acad. Sci. U.S.A.* 96 4832–4837 10.1073/pnas.96.9.4832 10220379 PMC21777

[B128] Zhu J. Winans S. C. (2001). The quorum-sensing transcriptional regulator TraR requires its cognate signaling ligand for protein folding, protease resistance, and dimerization. *Proc. Natl. Acad. Sci. U.S.A.* 98 1507–1512 10.1073/pnas.98.4.1507 11171981 PMC29287

[B129] Zottini M. Costa A. De Michele R. Ruzzene M. Carimi F. Lo Schiavo F. (2007). Salicylic acid activates nitric oxide synthesis in *Arabidopsis*. *J. Exp. Bot.* 58 1397–1405 10.1093/jxb/erm001 17317674

